# The effectiveness of a breastfeeding educational program on knowledge of antenatal women: A quasi-experimental study

**DOI:** 10.4314/ahs.v24i4.50

**Published:** 2024-12

**Authors:** Juliana Linnette D'Sa, Rasmia Alsomali, Ruqayyah Alhurubi, Rasha Assiri, Hanan Alobeid, Esraa Dandormah, Sahar Zamzam

**Affiliations:** 1 Maternal and Child Health Nursing Department, College of Nursing, King Saud University, Riyadh, Saudi Arabia; 2 East Jeddah Hospital, Saudi Arabia; 3 Riyadh Second Health Cluster, Saudi Arabia; 4 Khamis Mushait General Hospital, Saudi Arabia; 5 Qassim Health Cluster, Saudi Arabia; 6 Abha Maternity and Children Hospital, Saudi Arabia; 7 Faculty of Nursing, Alexandria University, Egypt

**Keywords:** Breastfeeding, educational intervention, knowledge, antenatal women, quasi-experimental study

## Abstract

**Background:**

Prenatal knowledge about breastfeeding is an important modulator of feeding practices.

**Objectives:**

This study aimed to evaluate effectiveness of a structured breastfeeding educational program on breastfeeding knowledge of antenatal women and identify factors that predict breastfeeding knowledge.

**Methods:**

A quasi-experimental pre-test post-test study was conducted between June and July 2022. Using convenience sampling,150 antenatal women from a tertiary care hospital in Riyadh, Saudi Arabia, were included. Personal and obstetrical information were collected using a demographic questionnaire. On Day one, the pre-test Breastfeeding Knowledge Questionnaire was administered, followed by the structured educational intervention. On Day 10, the post-test was conducted through telephonic interviews, using the same questionnaire. Descriptive statistics, paired t test and regression analysis were computed.

**Results:**

The mean post-test knowledge (24.97, SD=1.69) was significantly higher than the mean pre-test knowledge (20.26, SD=3.02), indicating that breastfeeding education was effective in improving the overall (p<0.001) and domain-wise knowledge: initiation and colostrum, exclusive breastfeeding, practices and techniques, nutritional aspects, and breastfeeding problems (p<0.001). High monthly income (OR=2.017; 95% CI=1.053–3.864; p=0.034), multigravidity (OR=3.117; 95% CI=1.489–6.525; p=0.003) and large family size (OR=2.889; 95% CI=1.479–5.643; p=0.002) were significant predictors of pre-test breastfeeding knowledge.

**Conclusion:**

The educational intervention was effective in improving breastfeeding knowledge in antenatal women. The findings can have significant implications for healthcare professionals seeking to enhance the delivery of breastfeeding information in clinical settings, particularly during the antenatal period.

## Introduction

Breastfeeding is an unparalleled way of providing ideal nutrition for the optimum growth and development of infants. It also has significant implications for maternal health^1^. The World Health Organization (WHO) and other international organisations recommend initiating breastfeeding within an hour of birth and exclusively breastfeeding infants for the first six months of life^2^.

However, despite these recommendations, global breastfeeding rates continue to decline. Global data recorded between 2015 and 2021 show that only 47% of infants were breastfed within an hour of birth; further, only 48% were exclusively breastfed, which is much lower than the global targets of 50% by 2025 and 70% by 2030. Moreover, rates of continued breastfeeding for 24 months have declined from 70% in the first year to 45% by the time the child is two years old, falling short of the global targets^3^. These figures emphasise that breastfeeding practices worldwide remain suboptimal.

In Saudi Arabia, breastfeeding practices are initially based on the recommendations mentioned in the Holy Quran, followed by those of global organisations^4^,^5^. Further, the country has officially adopted an infant and young child feeding policy^5^. However, breastfeeding practices in the country continue to remain suboptimal. Insufficient national data on breastfeeding makes it challenging to monitor practices in relation to global targets. A 2019 study reported that only 43.6% of mothers in Saudi Arabia successfully initiated breastfeeding within an hour of birth^4^. Conversely, another cross-sectional study conducted in the same year reported lower rates (13.9%)^6^. Various factors contribute to the deviation from international recommendations, including societal and cultural influences. A lack of accurate breastfeeding information also plays a role, leading to misconceptions. Women should have basic and accurate breastfeeding information to make informed decisions. The revised Baby-Friendly Hospital Initiative guidelines recommend discussing the importance and management of breastfeeding with pregnant women and their families (Step 3), considering it a key clinical practice enlisted under the Ten Steps of Successful Breastfeeding recommended by the WHO^7^.

Unfortunately, most antenatal education programmes lack sufficient information on breastfeeding. A review of 18 qualitative studies showed that mothers felt that infant feeding was insufficiently discussed during the antenatal period^8^. Women who do not receive adequate information during the antenatal period are at risk of engaging in poor breastfeeding practices. For instance, the risk of initiating mixed feeding before infants reached six months of age was reported among those who did not receive breastfeeding knowledge in antenatal clinics^9^. Contrastingly, those who received antenatal education followed optimal breastfeeding practices. Alshammari and Haridi found a positive association between mothers' exclusive breastfeeding knowledge and practices^10^. Adequate breastfeeding knowledge and positive attitudes are associated with early breastfeeding initiation and longer duration of breastfeeding^11^. Therefore, empowering pregnant women with accurate breastfeeding information could be one way of increase breastfeeding prevalence among the population.

Although the Ministry of Health of Saudi Arabia disseminates breastfeeding information to the public through its websites, this effort does not appear to be sufficient. Various scholars have emphasised the need to create breastfeeding awareness in Saudi Arabia through educational programmes^12^. Adequate and consistent information offered through organised educational programmes can equip women with adequate knowledge, skills, and positive attitudes necessary for successful breastfeeding, and improve their breastfeeding self-efficacy^13^,^14^. Although antenatal information can be provided in multiple ways, the traditional face-to-face individualised teaching method remains a viable option as it allows women to discuss their doubts and dispel misconceptions and myths about breastfeeding. Therefore, we aimed at evaluating a breastfeeding educational intervention for antenatal women, focusing on knowledge improvement and identifying factors that predict baseline breastfeeding knowledge.

## Methods

This study was conducted in a multidisciplinary tertiary care hospital in Riyadh, Saudi Arabia, using a one-group pre-test post-test quasi-experimental design. The sample size was 150. A post hoc power analysis calculated using G*power 3.1.9.2 indicated that the statistical power of the study was over 99%, with a large effect size at 5% significance level. Because of possibility of loss to follow up, 150 women were included in the study. Women in their third trimester who attended antenatal clinics or were admitted were recruited using convenience sampling. Inclusion criteria were: having at least a high school education, proficiency in Arabic, agreement to participate in the study, and no previous attendance of breastfeeding training sessions. Those for whom breastfeeding was contraindicated were excluded. Two self-reported Arabic questionnaires: an author-designed demographic questionnaire (DQ), which elicited responses on personal and obstetric variables, and a 26-item Breastfeeding Knowledge Questionnaire (BKQ) developed by Alsalamah (2018), were used for data collection^15^. The BKQ covered six content domains: initiation and colostrum, exclusive breastfeeding, practices and techniques, benefits, nutritional aspects, and breastfeeding problems. It included 15 True/False/Not sure response items and 11 single-response three option multiple-choice items; each correct response was assigned a score of ‘1’ and incorrect had a score of ‘0’. The overall score ranged from 0–26, with a corresponding percentage range of 0–100%; the scores were categorised as excellent knowledge (21–26; 80% or more), moderate knowledge (14–20; 51–79%), and poor knowledge (0–13; 50% or less). The original scale recorded robust methods of content validation, translation-back translation (English-Arabic), and good internal consistency reliability (Kuder Richardson-20=0.82).

### Intervention

The structured educational breastfeeding intervention developed by the authors addressed six content domains of the breastfeeding questionnaire. Following an extensive review of existing literature on breastfeeding, including recommendations from global organisations, the authors drafted a structured educational plan. The draft underwent content validation by three experts specialised in community health and maternity nursing, with expertise in breastfeeding teaching, clinical practice and research. Using a Likert type four point rating scale to assess the relevance and accuracy of the content, experts reached a unanimous agreement on the objectives, content, teaching methods, and allocated time, as outlined in the drafted educational plan. Based on one expert's suggestion, content on ‘breast engorgement’ and the time allotted were increased and the pre-final draft of the educational plan was translated to Arabic by a professional language expert, and then back-translated to English and reviewed by an expert proficient in both languages. The pre-final Arabic version was pre-tested during a pilot study involving 13 antenatal women and was found to be clear and feasible.

### Data collection

Data were collected between June and July 2022. On the first day, eligible participants were recruited for the study. The purpose and voluntary nature of participation, with the option to withdraw from the study at any time without any consequences, were explained to the participants. After signing the consent form, the DQ and BKQ (pre-test) were administered to them; thereafter, they returned the completed questionnaires to the researchers. A face-to-face individualised educational intervention was then conducted using the pre-validated structured educational plan. Theoretical content was presented through a lecture-discussion method using a PowerPoint presentation on an iPad; practical/skill components (breastfeeding techniques, including positioning and latching) were demonstrated using a dummy/doll. The skills were demonstrated again to ensure correct understanding by the participants. The total duration of the educational intervention was 30–40 minutes. On Day 10, the posttest was conducted through telephonic interviews (lasting 10–15 minutes) using the same BKQ as the pre-test; the interviewer recorded the responses in the questionnaire as the participants were not physically present at the hospital.

### Ethical considerations

The study was approved by the Institutional Review Board of King Saud University (No. E-21-652). Permission to use the BKQ was obtained from the author. Informed consent was obtained from the participants; they were aware that participation was voluntary and had the right to withdraw from the study. Confidentiality of the data was maintained.

### Statistical analysis

Frequencies, percentages, means, and standard deviations were computed. A paired t-test was performed on normally distributed data to determine differences in the mean overall and domain-wise pre-test and post-test knowledge scores. Binary regression analysis was performed to identify significant predictors of adequate pretest breastfeeding knowledge. The odds ratio and 95% CI were calculated, and a p-value of 0.05 was statistically significant. Data were analysed using the Statistical Package for Social Sciences (SPSS) version 26 (IBM Corp, Armonk, NY, USA).

## Results

### Demographic characteristics

Most participants were aged 30–34 years (38%), multigravidas (69.3%), between 34-40 gestational weeks (60.7%), had a bachelor's degree (60%), were homemakers (67.3%), and had a family size of more than three (56%). Most of their spouses (82.7%) worked in the government sector, and less than half (41.3%) had a monthly household income of 5,000–10,000 SR (1 SR = 3.77 US $ approximately); Table 1.

**Table 1:** Frequency and percentage distribution of demographic and obstetrical variables. (n=150)

Demographic and obstetrical variables		Frequency	Percentage
Age (in years)	18 - 24	9	6.0
	25 - 29	52	34.7
	30 - 34	57	38.0
	35 and above	32	21.3
Education	High school	36	24.0
	Diploma degree	8	5.3
	Bachelor's degree	90	60.0
	Master's degree and higher	16	10.7
Occupation	Employed	47	31.3
	Housewife/homemaker	101	67.3
	Student	2	1.3
Husband's employment	Business owner	2	1.3
	Government Employee	124	82.7
	Private Sector	22	14.7
	Unemployed	2	1.3
Family income	Less than 5000 SR	12	8.0
	5000 - 10000 SR	62	41.3
	10000- 15000 SR	57	38.0
	More than 15000 SR	19	12.7
Pregnancies	Multigravida	104	69.3
	Primigravida	46	30.7
Gestational age	28 - 34 weeks	59	39.3
	34 - 40 weeks	91	60.7
Family size	3 or less persons	66	44.0
	More than 3 persons	84	56.0

### Comparison of pre-test and post-test breastfeeding knowledge scores

In the pre-test, almost half of the participants recorded either excellent knowledge (49.3 %), moderate knowledge (48%), and 2.7% had poor knowledge. In the post-test, the proportion of participants with excellent knowledge increased to 96.7%, whereas those with moderate knowledge decreased (3.3%), and none (0%) had poor knowledge.

#### The research hypothesis

The study hypothesised that the antenatal women's mean post-test knowledge score will be significantly higher than that of their mean pre-test knowledge score. When tested using a paired t-test. Results showed a significant increase in the overall post-test mean score (M=24.97, SD=1.69) compared to the pre-test (M=20.26, SD=3.02), indicating the effectiveness of the breastfeeding educational intervention in improving the breastfeeding knowledge of antenatal women (p<0.001), and the research hypothesis was accepted (Table 2).

**Table 2:** Mean, Standard Deviation (SD) and t-value between Pre-test and Post-test knowledge **(n=150)**

Test	Mean	Mean Difference	SD	t	df	p-value^§^
Pre-test	20.26	4.71	3.09	18.639	149	.000***
Post-test	24.97					

§ p-value has been calculated for paired sample t-test

*** Significant at p<0.001 level

The breastfeeding knowledge domain-wise paired t-test showed a significant increase in the post-test means compared to the pre-test in all six knowledge domains (p<0.001), indicating the effectiveness of the educational intervention in improving the knowledge of participants in all knowledge domains; (Table 3).

**Table 3:** Domain-wise pre-test and post-test breastfeeding knowledge (n=150)

Breastfeeding knowledge domains	Pre-test		Post-test		p-value ^§^

	Mean	SD	Mean	SD	
1. Initiation and colostrum	2.54	0.57	2.94	0.26	<0.001 ***
2. Exclusive breastfeeding	2.98	0.86	3.82	0.51	<0.001 ***
3. Breastfeeding practices and technique	5.73	1.36	7.12	0.81	<0.001 ***
4. Advantages/benefits	4.38	0.83	4.84	0.41	<0.001 ***
5. Nutritional aspects of breastfeeding	1.82	0.45	1.96	0.18	<0.001 ***
6. Problems in breastfeeding	2.80	0.94	3.81	0.42	<0.001 ***

§ p-value has been calculated using paired sample t-test

*** Significant at p<0.001 level

### Factors predicting adequate breastfeeding knowledge

The predictors of adequate pre-test breastfeeding knowledge computed through binary linear regression (scores categorised as 1=21 or more and 0=<21) were multigravidity (OR=1.266; 95 % CI=1.489 - 6.525; p=0.003), high family monthly income (OR=2.017; 95% CI=1.053 - 3.864; p=0.034) and larger family size (more than three persons) (OR=2.889; 95% CI=1.479 – 5.643; p=0.002). However, interestingly, age, educational level, occupation, and gestational period did not predict excellent breastfeeding knowledge (p>0.05). Table 4.

**Table 4:** Regression analysis for the predictors of excellent pre-test breastfeeding knowledge in relation to demographic characteristics (n=150)

Factor	OR	95% CI	p-value
Age group			
<30 years	Ref		
=30 years	1.764	0.912 – 3.411	0.092

Educational level			
Diploma or high school	Ref		
Bachelor or higher	1.618	0.794 – 3.296	0.185

Occupation			
Employed/Student	Ref		
Unemployed	0.801	0.404 – 1.587	0.525

Monthly family income (Saudi Riyal)			
=10,000	Ref		
>10,000	2.017	1.053 – 3.864	0.034*

Number of pregnancies			
Primigravida	Ref		
Multigravida	3.117	1.489 – 6.525	0.003 **

Gestational week			
28 – 34 weeks	Ref		
34 – 40 weeks	1.266	0.656 – 2.442	0.482

Family size			
3 persons or less	Ref		
More than 3 persons	2.889	1.479 – 5.643	0.002 **

OR – Odds ratio; CI – Confidence Interval.

** Significant at p<0.01 level

* p<0.05 level

## Discussion

The findings of this study show that breastfeeding educational interventions was effective in improving antenatal women's overall and domain-wise breastfeeding knowledge. Factors that predicted excellent knowledge were multigravidity, high family income, and large family size. Our findings of improved breastfeeding knowledge after the educational intervention are similar to those reported previously by Hanafi et al.^14^ and other researchers^16^–^18^. For instance, Hanafi et al. demonstrated the effect of a 30-minute breastfeeding educational session on improving the knowledge and attitude of antenatal women in Saudi Arabia^14^. Similarly, in a Greek study, nulliparous women recorded a significant increase in breastfeeding knowledge after attending a four-hour midwifery-led breastfeeding educational intervention compared to those who did not receive the intervention^16^. The unique nature of our study was face-to-face individual teaching, allowing participants to clarify misconceptions and myths on the spot, thereby enhancing their breastfeeding knowledge. Although knowledge, skills, and attitudes towards breastfeeding are significant modulators of optimal breastfeeding practices, this study focused only on knowledge improvement. However, the findings can have significant implications for healthcare professionals seeking to enhance the delivery of breastfeeding information in clinical settings, particularly during the antenatal period. Thus, future studies evaluating educational interventions for breastfeeding should consider skills and attitudes as outcome variables.

Knowledge is an essential modulator of breastfeeding practices. It was observed that before the educational intervention, only approximately half (49.3%) of our participants recorded excellent knowledge. However, contrary to our findings, Alsalamah reported excellent breastfeeding knowledge in 72.6% of primiparous women in the same region using the same knowledge questionnaire^15^. The low pre-test knowledge in our participants compared to Alsalamah's study could be attributed to the differences in participant characteristics; one-third of our participants were primigravidae with no prior breastfeeding experience, as opposed to those with breastfeeding experience in Alsalamah's study^15^. Higher knowledge has been reported in mothers who had previously breastfed their infants than in those who had not^19^. Moreover, none of our participants had attended any breastfeeding teaching sessions before the intervention, which highlights the need for promoting breastfeeding knowledge from the antenatal period.

We found that pregnant women with a higher family income, multigravidity, and large family size had greater pre-intervention breastfeeding knowledge than their counterparts. Similarly, a previous cross-sectional study revealed that women with higher family income had better knowledge regarding breastfeeding practices^19^. Generally, those with a higher income have better access to health-related educational materials, including internet resources, and consequently, better opportunities to seek breastfeeding information. Similar to our findings, research has previously shown that multigravidity and larger family size are predictors of good breastfeeding knowledge. A higher number of pregnancies also improved the knowledge of the participants. A Saudi study found higher levels of breastfeeding knowledge among mothers with five children or more^12^. Another study conducted in the United Arab Emirates reported better knowledge among women living in large families with their husbands, children, and relatives^20^; women may have received informal breastfeeding information from their own mothers and other family members, thereby enhancing their knowledge despite not having received any formal breastfeeding education.

We found that age, education, occupation, and gestational period were not associated with better knowledge, which is consistent with a previous report^21^. Contrarily, higher breastfeeding knowledge was found in those with higher education^19^,^20^, older age^22^, and those who were employed^19^. The mixed findings regarding these factors call for further studies with larger sample sizes which may lend further support to the fact that these factors vary in different populations.

The strength of this study was the individualised face-to-face method used to educate antenatal women, providing an opportunity to clarify misconceptions and myths. Nevertheless, the study has certain limitations. Selecting a sample through convenience sampling from a single hospital may have limited the generalisability of the findings. Futhermore, we included women who had at least a high school education, thereby limiting the generalizability to those educated. Future research efforts should not only focus on more diverse samples but also examine the effect of the intervention on women's actual behaviour through follow-up studies using a control group.

## Conclusion

The findings suggest that individualised face-to-face educational interventions are effective in improving breastfeeding knowledge among antenatal women. Further research is needed to determine whether such interventions are useful for knowledge retention and the transfer of knowledge into practice during the postnatal period and beyond. Furthermore, healthcare professionals can play an important role in improving breastfeeding knowledge and skills during pregnancy, thereby promoting optimal breastfeeding practices.
